# Primary Papillary Mucinous Adenocarcinoma of the Ureter Mimicking Genitourinary Tuberculosis

**DOI:** 10.4061/2010/738407

**Published:** 2010-06-16

**Authors:** Hanni Gulwani, Aruna Jain

**Affiliations:** Department of Pathology, Bhopal Memorial Hospital & Research Centre, 91-755-462038, Bhopal, India

## Abstract

Primary adenocarcinomas of the renal pelvis and ureter are rare and account for less than 1% of all malignancies at this site. We report a case of primary papillary mucinous adenocarcinoma of the ureter that clinically mimicked genitourinary tuberculosis. Early diagnosis is important for the better outcome.

## 1. Introduction

Primary adenocarcinomas of the renal pelvis and ureter are rare and have been reported mainly in the form of single cases or small series. Most of these arise as a result of glandular metaplasia. The predisposing factors include nephrolithiasis and repeated infections. Associated malignancies are high grade and widely invasive at presentation [1]. We hereby present an unusual case report of primary papillary mucinous adenocarcinoma of the ureter that clinically mimicked genitourinary tuberculosis. 

## 2. Case Report

 A 54-year-old male patient presented to the Urology Department of Bhopal Memorial Hospital and Research Centre with right flank pain for last two weeks. There were no complaints of hematuria and pyuria.

## 3. Radiological Investigations

 Ultrasound examination revealed moderately hydronephrotic right kidney with dilated right ureter. The dilatation involved lower 1/3rd of the ureter suggesting stricture. No calculus or mass was seen in the right kidney. Bulb ureterogram revealed complete ureteric block. Renal function tests were deranged. On LUT endoscopy, the right ureteric orifice, urethra, bladder neck, and bladder were within normal limits. The left kidney and ureter were also unremarkable.

A clinical diagnosis of genitourinary tuberculosis associated with right-sided hydronephrosis and hydroureter was made. Perioperatively, right ureteric lumen was narrowed and occluded in its lower one third. The wall was mildly edematous. Patient refused to undergo nephrectomy; therefore, the stricture was relieved and ureteric reimplantation was achieved using Boari flap with psoas hitch. Biopsy was taken from right ureteral stricture and sent for histopathological examination. 

## 4. Pathology Findings

**Figure 1:**
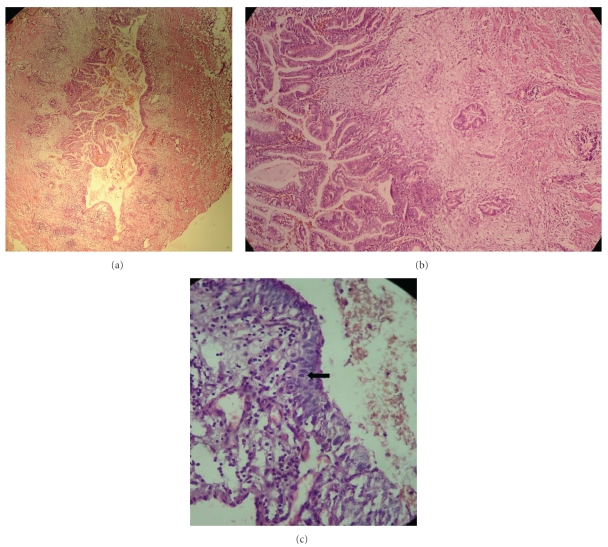
(a) Papillary mucinous adenocarcinoma of the ureter. Nearly two-thirds of the ureteric lumen were occupied by the tumor. (b) Higher magnification shows papillary cores covered by tall columnar epithelium. Tumor is seen infiltrating the ureteric wall in form of glands. (c) Higher magnification showing dysplastic ureteric epithelium with increased mitotic figures (marked with arrow).

**Figure 2:**
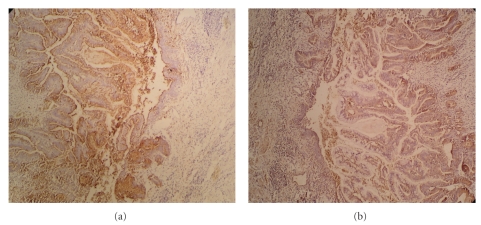
(a) Immunohistochemical stain. Tumor cells stain positive for CEA. (b) Immunohistochemical stain. Tumor cells stain positive for CA 19.9.

 Routinely stained sections from the ureteric biopsy revealed a papillary adenocarcinoma filling the lumen. The tumor was occupying nearly two thirds of the ureteric mucosa and rest one third of the ureteric mucosa was lined by attenuated transitional epithelium with focal ulceration (Figure 1(a)). The tumor had papillary projections with a central fibrovascular core. The papillae were lined by tall columnar stratified epithelium and several of the epithelial cells contained mucin. In other areas tumor had glandular pattern and the glands were infiltrating the lamina propria, muscle layer and reaching up to the serosa (Figure 1(b)). The adjacent ureteric epithelium showed dysplastic changes (Figure 1(c)). On immunohistochemical staining, the tumor cells stained positive for CEA, CA19.9 (Figures 2(a) and 2(b)), CK 7, and CK 20.

## 5. Discussion

Primary adenocarcinoma of the renal pelvis and ureter is a rare tumor and constitutes less than 1% of the malignancies arising from ureter and renal pelvis. Most of the previous case reports have been from western literature and this malignancy is still rarer in people of Asian origin. Genitourinary tuberculosis is fairly common in developing countries and presents as ureteric stricture in 9% of cases [2]. The present case was also clinically misinterpreted as genitourinary tuberculosis. 

In our patient, the presentation was with colicky pain and on further investigations he was found to have chronic pyelonephritis and a stricture in the lower third of right ureter. The patient did not have any hematuria or stones. The possible cause of intestinal metaplasia in the present case could be attributed to recurrent urinary infections that lead to pyelonephritis. Further exploration of the right kidney and pelvis did not reveal any associated malignancy in the urinary tract. Additionally, the adjacent ureteric epithelium showed dysplastic changes with presence of prominent mitotic figures.

The postoperative period was uneventful. Bone scan was done later to look for metastases and it showed multiple metastases in the vertebral column. Extensive investigations were done to rule out any primary malignancy. Abdominal and chest CT scan, Pelvic ultrasound, intravenous urography, and GI exploration did not reveal any other primary focus. Since the patient refused to undergo any surgery, he was referred to cancer hospital for further treatment. He was given systemic chemotherapy followed by irradiation. Bone and CT scan done after treatment showed complete response to therapy. 

Renal pelvic and ureteral tumours occur simultaneously with each other in 6–38% of cases and 50% of individuals with upper collecting system. Lesions can later on develop bladder tumours [3]. Adenocarcinoma at this site has been subdivided into tubulovillous, mucinous, papillary nonintestinal, and signet-ring cell type [4].

Most of these malignancies occur in adults but pediatric cases have also been reported. Calculi and repeated infections are the common predisposing factors that lead to mucinous metaplasia. According to a report by Rao et al. [5] enteric-type adenocarcinoma of upper tract urothelium was associated with ectopic ureter and renal dysplasia. They concluded that closed-spaced nonfunctional urothelium can also lead to malignant degeneration. 

A papillary architecture and resemblance to mucinous adenocarcinoma of colon are common. In our present case, immunohistochemical analysis showed strong expression for CA 19.9 in tumor cells. The immunomarker was also seen in the adjacent normal transitional cell epithelium of ureter [6]. In a case report by Hosomi et al. primary adenocarcinoma of the ureter had hepatoid areas and contained bile pigment [7]. The tumor also showed immunohistochemical expression of alpha-fetoprotein.

It is important to differentiate the primary adenocarcinomas of ureter from other gland-forming malignancies arising from the transitional epithelium as the former one is associated with grave prognosis. The differential diagnosis includes transitional cell carcinoma with glandular metaplasia, muci-urothelial cancer, microcystic transitional cell cancer, and transitional cell cancer with mucoid cytoplasmic inclusions. It is also essential to rule out the possibility of metastatic adenocarcinoma in ureter before labeling the tumor as primary in origin. 

To conclude, although rare, primary adenocarcinoma of the ureter is a known entity and presents commonly as ureteric stricture. Histological analysis is thus essential to establish the diagnosis.
